# Editorial: Fundamentals and Advances in Cardiopulmonary Rehabilitation

**DOI:** 10.3389/fresc.2026.1875363

**Published:** 2026-05-28

**Authors:** Iván Rodríguez-Núñez

**Affiliations:** Human Movement Sciences Research Laboratory (LICiM-UdeC), Department of Kinesiology, University of Concepción, Concepción, Chile

**Keywords:** autonomic regulation, cardiopulmonary rehabilitation, dyspnea, exercise adherence, precision rehabilitation therapy, transcranial magntic stimulation

**Editorial on the Research Topic**
Fundamentals and advances in cardiopulmonary rehabilitation

Chronic cardiovascular and respiratory diseases remain among the leading causes of morbidity and mortality worldwide, exerting a profound impact on functional capacity, quality of life, and healthcare systems (1). In this context, cardiopulmonary rehabilitation has emerged as a cornerstone non-pharmacological intervention, consistently demonstrating its capacity to improve physiological function, reduce symptoms, and enhance long-term outcomes (2). However, despite its well-established efficacy, the field continues to face important challenges related to mechanistic understanding, intervention optimization, and real-world implementation.

The contributions included in this Research Topic can be conceptually organized into three interconnected domains: strategies targeting central and autonomic regulation, functional outcomes and recovery trajectories, and implementation and adherence challenges. Together, these domains reflect a progressive transformation in rehabilitation, moving beyond traditional exercise-based approaches toward integrative, multidimensional, and person-centered models that recognize the patient as an embodied and experiential subject (3, 4).

A first domain encompasses rehabilitation strategies targeting central and autonomic regulation, integrating both emerging technologies and structured exercise interventions through their shared effects on neural and autonomic processes. The narrative review by Xu et al. on transcranial magnetic stimulation introduces a perspective in which respiratory dysfunction is not solely interpreted as a peripheral or mechanical impairment, but as a phenomenon rooted in cortical and subcortical processes. By modulating corticospinal excitability, influencing neurotransmitter dynamics, and interacting with limbic circuits involved in emotional processing, transcranial magnetic stimulation provides emerging evidence that targeting central neural mechanisms may lead to clinically meaningful outcomes, including the attenuation of dyspnea through altered respiratory drive and perceptual processing.

From a complementary yet mechanistically distinct perspective, the systematic review and meta-analysis by Hu et al. on Baduanjin exercise demonstrates that low-to-moderate intensity, breathing-centered movement practices can induce significant improvements in functional capacity and cardiovascular function. Importantly, these interventions extend beyond peripheral adaptations by engaging autonomic and emotional regulation through coordinated breathing, attention, and movement. Despite differing mechanisms, both approaches converge on the principle that clinically relevant outcomes are partly mediated by central and autonomic processes. This supports the view that symptom perception, particularly dyspnea, is not solely determined by peripheral physiological constraints but is also shaped by central and integrative regulatory mechanisms. Collectively, these findings reinforce a broader conceptual shift toward rehabilitation models that integrate neurobiological, physiological, and experiential dimensions.

A second domain addresses functional outcomes and recovery trajectories, providing insight into the longitudinal dynamics of rehabilitation. Goulart et al. conducted a 12-month follow-up study in individuals recovering from COVID-19, demonstrating significant improvements across multiple cardiopulmonary exercise testing parameters, including peak oxygen uptake (VO₂peak), ventilatory efficiency (VE/VCO₂ slope), and oxygen uptake efficiency slope (OUES), alongside enhancements in pulmonary function and inspiratory muscle strength. Complementing this longitudinal perspective, Kouda et al. illustrate how cardiopulmonary exercise testing can guide individualized rehabilitation, showing that physiologically informed interventions can actively modulate recovery trajectories and restore functional independence. Extending this evidence base, Xiangli et al. provide a systematic review with meta-analysis of studies in individuals with spinal cord injury, demonstrating that functional electrical stimulation significantly improves key expiratory parameters, including peak expiratory flow, maximal expiratory pressure, and forced vital capacity, while also enhancing aerobic capacity when combined with exercise modalities. Taken together, these studies illustrate two complementary dimensions of recovery: the natural trajectory of physiological restoration and the capacity to actively modulate this trajectory through targeted, physiologically informed interventions.

The third domain emphasizes implementation and adherence challenges, a critical yet often underexplored dimension of rehabilitation science. Although studies focusing on functional recovery trajectories demonstrate the physiological capacity for improvement, evidence on rehabilitation adherence highlights a critical translational gap: patients’ ability to engage with and sustain these interventions in real-world contexts (5). In this regard, Feng et al. investigated the relationship between kinesiophobia and exercise adherence in older adults with chronic obstructive pulmonary disease, identifying a strong negative association and a clinically relevant non-linear threshold effect, whereby adherence declines sharply once fear surpasses a critical level. Complementing this behavioral perspective, Hu et al. examined postoperative pulmonary rehabilitation compliance among patients with lung cancer, demonstrating moderate adherence and identifying key determinants such as advanced age, lower educational level, rural residence, lower income, and limited social support. Extending this perspective to the healthcare system, Tang et al. showed that gaps in knowledge, attitudes, and clinical practices among nurses significantly influence the implementation of oxygen therapy, underscoring the importance of professional training and institutional support. Taken together, these findings indicate that the effectiveness of cardiopulmonary rehabilitation is not determined solely by intervention efficacy, but by the interaction between psychological readiness, socioeconomic context, professional competence, and system-level capacity to support sustained patient engagement.

Taken together, the studies included in this Research Topic illustrate a field undergoing significant transformation. Cardiopulmonary rehabilitation is evolving from a predominantly exercise-based discipline into a complex, integrative science that incorporates neurophysiology, behavioral medicine, and health systems research. This evolution reflects a broader paradigm shift toward precision and integrative rehabilitation, where interventions are tailored not only to physiological impairments but also to individual, social, and contextual factors (6, 7). Future research should focus on aligning mechanistic insight, individualized intervention, and real-world implementation, ensuring that advances in cardiopulmonary rehabilitation translate into meaningful and sustained patient benefit.
